# Redox dyshomeostasis modulation of the tumor intracellular environment through a metabolic intervention strategy for enhanced photodynamic therapy

**DOI:** 10.7150/thno.75837

**Published:** 2022-08-15

**Authors:** Zuo Yang, Chaoqiang Qiao, Qian Jia, Zhuang Chen, Xiaofei Wang, Xuelan liu, Ruili Zhang, Kanyi Pu, Zhongliang Wang

**Affiliations:** 1 Lab of Molecular Imaging and Translational Medicine (MITM), Engineering Research Center of Molecular and Neuro Imaging, Ministry of Education, School of Life Science and Technology, Xidian University & International Joint Research Center for Advanced Medical Imaging and Intelligent Diagnosis and Treatment, Xi'an, Shaanxi 710126, China.; 2 Academy of Advanced Interdisciplinary Research, Xidian University, Xi'an, Shaanxi, 710071, China.; 3 School of Chemical and Biomedical Engineering, Nanyang Technological University, 70 Nanyang Drive, Singapore, 637457, Singapore.

**Keywords:** photodynamic therapy, metal-organic framework, metabolic pre-intervention, hypoxia, glutathione

## Abstract

**Rationale:** Photodynamic therapy (PDT) is a clinically approved anticancer treatment with a promising therapeutic prospect, however, usually suffers from the unfavorable intracellular environment including cellular hypoxia and excessive glutathione (GSH). Comprehensive and long-term modulation of tumor intracellular environment is crucial for optimizing therapeutic outcomes. However, current strategies do not enable such requirements, mainly limited by flexible networks of intracellular metabolic avenues.

**Methods:** A metabolic pre-intervention (MPI) strategy that targets critical pathways of cellular metabolism, ensuring long-term modulation of the intracellular environment. A versatile lipid-coating photosensitive metal-organic framework (MOF) nano-vehicle encapsulating aerobic respiration inhibitor metformin (Met) and GSH biosynthesis inhibitor buthionine sulfoximine (BSO) (termed PBMLR) was developed for comprehensive sustainable hypoxia alleviation and GSH downregulating.

**Results:** Since MPI could effectively circumvent the compensatory accessory pathway, PBMLR, therefore functioned as an efficient singlet oxygen (^1^O_2_) radical generator during the subsequent laser irradiation process and enhanced PDT anti-tumor efficiency. We emphasized the concordance of long-term hypoxia alleviation, persistent GSH depletion, and tumor enrichment of photosensitizers, which is very meaningful for a broad therapeutic time window and the successful enhancement of PDT.

**Conclusion:** Our findings indicate that maintaining the sensitivity of tumor cells via MPI could enhance anti-tumor PDT, and may be applied to other dynamic therapies such as radiodynamic therapy and sonodynamic therapy.

## Introduction

As a widely recognized and minimally invasive cancer treatment, photodynamic therapy (PDT) has achieved great success in clinical practice. However, the tumor intracellular environment, which limits singlet oxygen (^1^O_2_) efficiency, is not conducive to PDT 1-3. An unfavorable tumor intracellular environment formed by cancer-specific alterations in metabolism 4 is mainly represented by insufficient oxidation and excess reduction. Specifically, the adaptive upregulation of antioxidant systems, such as glutathione (GSH), can rescue cancer cells from high levels of reactive oxygen species (ROS) during PDT 5. Moreover, high levels of aerobic respiration, adapted to proliferation, further reduce the amount of intracellular oxygen available for PDT, leading to lower photo-induced ROS accumulation 6. Together, these two major features of the tumor intracellular environment hinder the clinical application of PDT in solid tumor treatment.

To remodel the tumor intracellular environment, well-designed oxidant-based nanomaterials used as oxygen donors, such as Cu(II) 7, and/or sacrificial oxides for GSH scavenging, such as MnO_2_ 8, have been developed in the past decade. However, efforts to supply oxygen through hydrogen peroxide (H_2_O_2_)-catalyzed reactions make it difficult to maintain a constant oxygen supply. This is limited by unsustainable intracellular H_2_O_2_ concentrations (below 50 μM) 9 and continuous surrounding oxygen consumption during mitochondrial respiration 10,11. Similarly, GSH depletion strategies do not provide long-lasting effects, as low levels of GSH trigger the antioxidant protection system to compensate for insufficient GSH via multiple routes 12,13. Such transient regulation leads to limited enhancement in PDT efficiency. Moreover, it cannot provide a long therapeutic time window. Targeting cellular metabolism is a promising approach for overcoming dysregulated intracellular microenvironments involved in therapeutic resistance 14,15. Therefore, we envisioned an efficient and durable intervention of the intrinsic metabolic pathway to achieve sustainable intracellular environment remodeling to facilitate the effect of PDT.

**Scheme 1:**
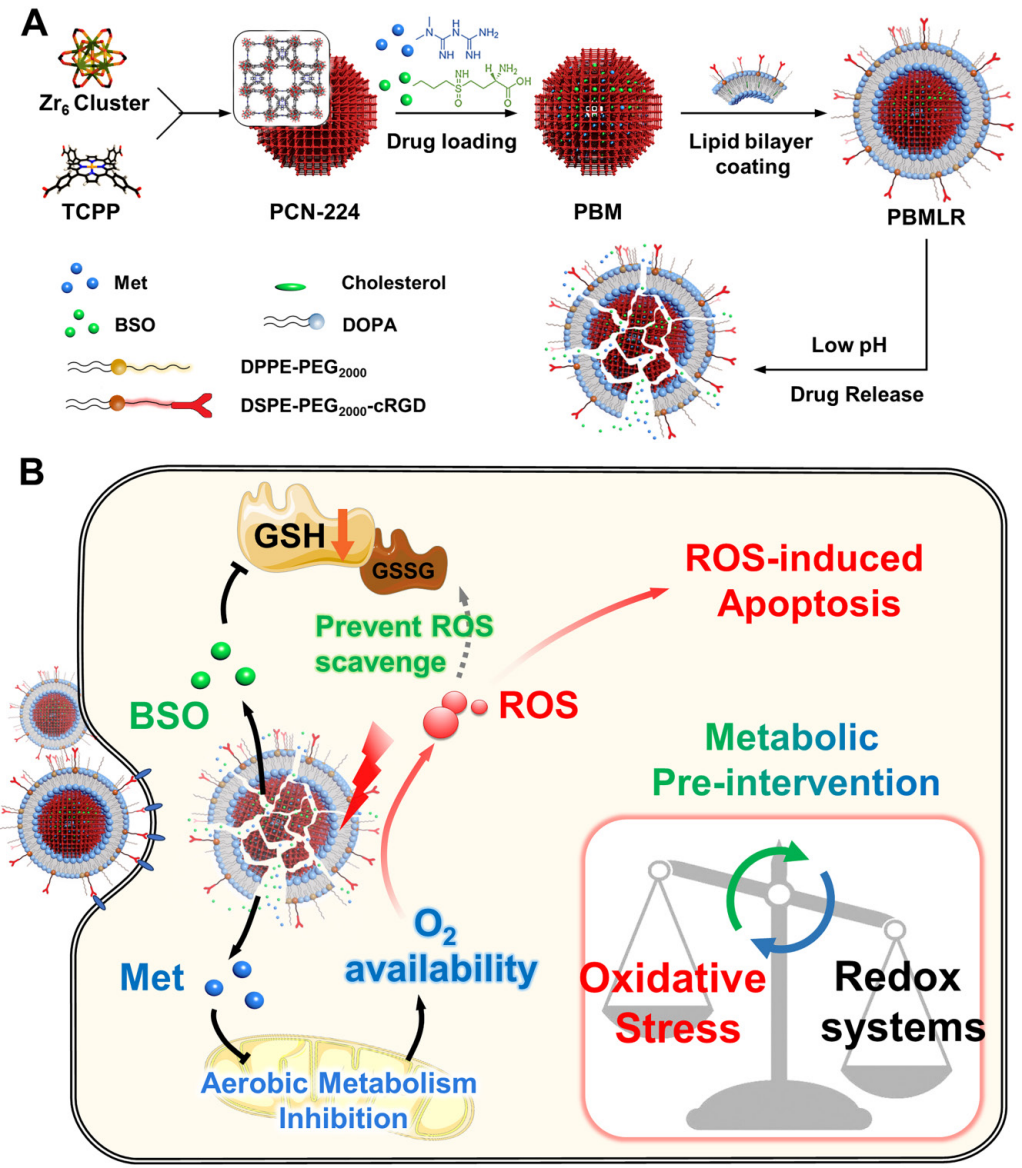
The design and function of PBMLR. (A) Construction of the porphyrinic metal-organic framework nanoparticle PBMLR through drug loading and lipid bilayer coating. (B) Metabolic pre-intervention model of PBMLR in tumor cells. After uptake of PBMLR nanoparticles via cRGD recognition sites, two inhibitor molecules release and play a synergistic role in metabolic intervention. Met inhibits aerobic metabolism, leading to the accumulation of oxygen. Meanwhile, BSO inhibits GSH biosynthesis, leading to a weakened detoxification pathway to prevent the loss of exogenous ROS. These modulations adjust the balance of intracellular redox homeostasis and will eventually induce cell death by enhancing the effect of PDT.

To suppress oxygen consumption, metformin (Met) was chosen as a cell respiration-inhibiting drug. This drug can induce a substantial reduction in cellular oxygen consumption rate and is less likely to induce lactic acidosis than other regulators 16. Buthionine sulfoximine (BSO) was chosen as a γ-glutamylcysteine synthetase (γ-GCS) inhibitor, which can block GSH replenishment by inhibiting the biosynthetic route at the source 17. Therefore, we described a metabolic pre-intervention (MPI) strategy toward the intrinsic metabolic pathway based on a nano-vehicle consisting of a PDT skeleton and inhibitor molecules (Scheme 1A). The application of the nanoplatform was aimed at specifically delivering metabolic regulators for effective anti-tumor PDT treatment. Porous photosensitizer coordination network (PCN-224) nanoparticles (NPs) were selected as versatile nano-vehicles, owing to their good photodynamic activity and sustainable cargo release ability. The NPs were further functionalized with a lipid bilayer consisting of the RGD peptide using a simple phospholipids coating method (Scheme 1A), described in our previous study 18,19. The NPs exhibited cancer and neovasculature targeting ability while ensuring stable circulation in the bloodstream *in vivo*. This nanoparticle construct was termed PBMLR. After PBMLR was internalized by integrin α_v_β_3_-overexpressed tumor cells, two inhibitor molecules were sustainably released and played a synergistic role in long-term microenvironment remodeling, leading to the increased sensitivity of tumor cells to PDT (Scheme 1B). Moreover, the tumor enrichment of photosensitizer, long-term hypoxia alleviation, and persistent downregulation of GSH induced by PBMLR lasted 24 h, providing a broad therapeutic time window for PDT laser irradiation after a single intravenous infusion of PBMLR. Hence, this PBMLR-mediated MPI strategy provides a new paradigm for long-term intracellular environment modulation that amplifies the effects of ROS-based dynamic therapies.

## Materials and methods

### Reagents and instrumentation

All chemical reagents, if not specified, were purchased from Sigma-Aldrich and used without purification. 1,2-dioleoyl-sn-glycerol-3-phosphate, sodium salt (DOPA), N-(Carbonyl-methoxypolyethylene O-glycol 2000)-1,2-dipalmitoyl-sn-glycerol-3-phosphoethanolamine (DPPE-PEG 2000), and 1,2-dipalmitoyl-sn-glycero-3-phosphoethanolamine-N[methoxy(polyethyleneglycol)-2000], ammonium salt (DSPE-PEG 2000) were purchased from Avanti Polar Lipids. Cyclo (Arg-Gly-Asp-d-Phe-Cys) c(RGDfC) was purchased from CHINAPEPTIDES CO., LTD. 1,1-dioctadecyl-3,3,3,3-tetramethylindodicarbocyanine perchlorate (DiD) and 1,1-dioctadecyl-3,3,3,3-tetramethylindotricarbocyanine iodide (DiR) were purchased from AAT Bioquest. Sulfosuccinimidyl 4-(N-maleimidomethyl)cyclohexane-1-carboxylate (SMCC) was purchased from Alfa. Met was purchased from Ark pharm. ROS assay kit (DCFH-DA) and GSH assay kit were purchased from the Beyotime Institute of Biotechnology (China). Singlet oxygen sensor green (SOSG) was purchased from Thermo Fisher Scientific. BALB/c female mice were supplied by the Animal Center of the Fourth Military Medical University (Xi'an, China). Animal protocols related to this study were reviewed and approved by the Institutional Animal Care and Use Committee of the Fourth Military Medical University (approval number: 20190229). The crystal structure of the samples was determined by powder X-ray diffraction (PXRD) patterns (Bruker D8, Germany). The samples' hydrodynamic diameter and Zeta potential were performed on a Malvern Zeta Sizer Nano (Malvern Instruments). Transmission electron microscopy (TEM) images were obtained using a JEM-2100F electron microscope. Confocal fluorescence imaging experiments were obtained using confocal laser scanning microscopy (Leica TCS SP5). Absorbance in 3-(4,5-dimethylthiazol-2-yl)-2,5-diphenyltetrazolium bromide (MTT) assay was measured in a Multiskan (Thermo Scientific). The Live animal imaging system (IVIS Lumina III, US) was applied to *in vivo* imaging.

### Synthesis of PCN-224

The porphyrin-based MOF nanoparticle, PCN-224, was synthesized according to the literature 20. Briefly, H2TCPP (10 mg, 0.013 mmol), ZrOCl_2_·8H_2_O (30 mg, 0.093 mmol), and benzoic acid (280 mg, 2.3 mmol) were added to 14 mL of DMF in a 50 mL Teflon-lined stainless-steel autoclave at 90 °C for 5 h. After cooling down to room temperature, PCN-224 nanoparticles were collected by centrifugation followed by washing with fresh DMF and ethanol three times. The resulting PCN-224 nanoparticles were suspended in ethanol for further characterization and analysis.

### Loading of Met and BSO into PCN-224

To prepare met-loaded nanoparticles, met was dissolved in deionized water and added into naked PCN-224 aqueous suspension dropwise with a weight ratio of 1:3; meanwhile, ultrasonic dispersed in darkness at room temperature for 30 min. The Met@PCN-224 nanoparticles (PM) were collected by centrifugation at 12000 g for 10 min and washed with deionized water twice to remove the free Met. The preparation methods of the BSO@PCN-224 nanoparticles (PB) and Met-BSO@PCN-224 nanoparticles (PBM) are the same as those mentioned above.

### Surface functionalization of nanoparticles with the lipid bilayer

A total of 4 mg of DOPA/cholesterol/DPPE-PEG2000/DSPE-PEG2000-cRGD (mass ratio of 15:5:5:2) were dissolved in 1 mL of chloroform. Then, the organic solvent was removed using a rotary evaporator at 42 °C and dried under a vacuum overnight. The DiD or DiR labeled lipid bilayer was prepared with an additional 72 μL of 10 mg mL^-1^ DiD or DIR according to the above procedure. The raw lipid bilayer was prepared without the cRGD ligand portion. Following the addition of PCN-224 aqueous suspension (6 mL, 0.5 mg mL^-1^) to the formed lipid film, probe sonication was immediately used for 30 min. The obtained PCN-224@Lipid-RGD nanoparticles (PLR) were washed with deionized water three times, collected by centrifugation (10000 rpm, 10 min), and resuspended in deionized water, medium, or PBS as indicated. The functionalization methods of the PM@lipid-RGD (PMLR), PB@Lipid-RGD (PBLR), and PBM@Lipid-RGD (PBMLR) are the same as those mentioned above.

### Drug loading and release

The drug loading efficiency (LE) was calculated by the equation:

LE = (m_drug_/m_PBMLR_) × 100%

Where, m_drug_ represents the weight of Met or BSO loaded in the PBMLR, respectively. To determine the loading efficiency, the PM, PB, and PBM NPs were dissolved by PBS, respectively, and then met was quantified by high-performance liquid chromatography (HPLC). For the pH-dependent drug release, PBMLR NPs were dispersed in PBS with different pH, and the released Met and BSO were determined by HPLC at different time intervals.

To quantify the drug release, 5 mL of 1 mg mL^-1^ PBMLR was added and sealed in a dialysis bag. The bag was immersed in PBS buffer solution with different pH values (pH = 7.4 or 5.5). Dialysis was preceded under constant stirring (200 rpm) at 37 °C. Then, 1 mL of dialysis solution was collected at different time points (1, 2, 4, 8, 12, 24, 36, and 48 h). Each sample solution was digested in 1 mL of 68% nitric acid for 1 h at 100 °C. Then, the solutions were allowed to cool and diluted to 10 mL by using ultrapure water. The diluted solutions were used for inductively coupled plasma mass spectrometry analysis.

### Determination of ^1^O_2_

Singlet oxygen sensor green (SOSG) assay was used to determine the generation of ^1^O_2_. Briefly, 5 mM SOSG working solution was prepared by dissolving 100 μg SOSG into 33 μL methanol. And 10 μL of SOSG working solution was mixed with 500 μL of 100 μg mL^-1^ PBMLR. Then, the mixed solution was irradiated continuously by a 660 nm laser, and the fluorescence intensity (λ_Ex_ = 504 nm/λ_Em_ = 550 nm) of the mixed solution was recorded every minute for 25 min. The solution contained 500 μL of deionized water and 10 μL of SOSG working solution was used as the control group.

### Cell experiments

4T1 cells were cultured in RPMI-1640 medium with 10% FBS and 1% penicillin/streptomycin. MCF-7 and U87 cells were cultured in DMEM with 10% FBS and 1% penicillin/streptomycin. All cells were cultured at 37 °C in a humidified 5% CO_2_ atmosphere.

### Cytotoxicity assay

4T1 cells were seeded in 96-well plates at a density of 5 × 10^3^ cells per well for 24 h and then treated with different concentrations (0, 10, 25, 50, 75, and 100 µg mL^-1^, as an equivalent dosage of MOF) of PBMLR. After 24 h incubation, cell viability was evaluated by using the MTT assay.

### Cellular uptake

Cell uptake of PBMLR was determined by flow cytometry and fluorescence image. 1 × 10^5^ cells were seeded in each well of a 6-well plate and incubated for 12 h. Afterward, 100 μL solution with different concentrations of the nanoparticles was added to each well. The cells were incubated for 12 h and washed with PBS to remove free materials. For flow cytometry, 200 μL of 0.5% trypsin-EDTA solution was introduced to detach cells and 1 mL of culture media containing 10% FBS was used to terminate digestion and re-disperse cells. The cells were centrifugated and dispersed with PBS. The fluorescence signal of cells was collected by flow cytometry. For fluorescence images, the images of cells in a 6-well plate were observed by the inverted fluorescence microscope (λ_Ex_ = 640 nm/λ_Em_ = 670 nm).

### Cellular GSH assay

4T1 cells were seeded into a 6-well plate at a density of 1 × 10^5^ cells per well and cultured for 24 h. After incubation with different concentrations of BSO, PBLR, or PBMLR in separate hours, cells were harvested and analyzed by GSH assay kit according to the manufacturer's protocol.

### Intracellular ROS generation

4T1 cells at the logarithmic phase were collected and seeded in a 6-well plate and incubated for 12 h. Then, different concentrations of materials were added to each well. After incubated for 6 h, the cells were irradiated with 660 nm laser for 10 min and washed with RPMI-1640 culture media to remove free materials. DCFH-DA working solution was added into wells and incubated for 20 min. Afterward, the cells were washed with PBS and the fluorescence intensity of cells (λ_Ex_ = 488 nm/λ_Em_ = 525 nm) was observed by the inverted fluorescence microscope.

### Photoinduced cytotoxicity

4T1 tumor cells were seeded in 96-well plates at a density of 5 × 10^3^ cells per well. After 24 h incubation, cells were treated with PBS, PLR, PBLR, PMLR, or PBMLR for 12 h, then washed with PBS, and each well was added 100 µL fresh medium. Cells were then irradiated using a 660 nm light-emitting diode (LED) at 10, 20, 50, or 100 mW cm^-2^ for 10, 15, or 20 min separately. After 12 h further incubation, cell viability was measured by MTT assay. For live/dead cell staining and apoptosis analysis, 4T1 cells were treated and irradiated at 50 mW cm^-2^ as described above. At 2 h after irradiation, cells were either stained with Calcein-AM and PI for 10 min, washed with PBS, and observed by confocal microscopy, or stained with annexin V-FITC and PI and analyzed by flow cytometry.

### *In vivo* experiments

To establish the mouse tumor model, 100 μL of 5 × 10^5^ cells 4T1 cells were injected subcutaneously into the right hind limb of each 4-week-old female BALB/c mouse. The tumor-bearing mice were used when the tumor volume reached 100 mm^3^ (for imaging) or 70 mm^3^ (for the experiment of therapy).

### *In vivo* tumor targeting

Tumor-bearing mice were randomly divided into two groups (n = 6) and intravenously injected with DIR-labeled PBML (without cRGD modification) or PBMLR nanoparticles (equivalent MOF dosage 10 mg kg^-1^), respectively. After narcotizing with isoflurane, the fluorescence images of mice were captured using the IVIS system at different time points (0, 1, 4, 12, 24, and 48 h). Subsequently, the mice were sacrificed and the major organs (including heart, liver, spleen, lung, kidney, and brain) and tumors were harvested for *ex vivo* imaging.

### *In vivo* therapeutic experiments

Tumor-bearing mice were randomly divided into six groups (n = 6) for the following treatments: 1) saline, 2) PBMLR, 3) PLR with laser irradiation, 4) PBLR with laser irradiation, 5) PMLR with laser irradiation, and 6) PBMLR with laser irradiation. The equivalent doses of MOF, Met, and BSO in each injection were 10, 6.3, and 5.35 mg kg^-1^, respectively. For mice that received PDT, at 24 h post-injection, the animals were exposed to a 660 nm LED with the irradiance of 50 mW cm^-2^ for 15 min. The body weight and tumor volume of the mice were monitored every 2 days during the whole experiment. Mice were euthanized on day 14 since the treatment started for further investigation.

### Statistical analysis

All the data were examined statically using the SPSS17.0 statistical analysis software. All the error bars indicated mean ± standard deviation. Student t-test or one-way analysis of variance (ANOVA) followed by Tukey's honestly significant difference (HSD) test was applied for comparison between two groups or among multiple groups, respectively, as indicated * *P* < 0.05, ** *P* < 0.01, and *** *P* < 0.001.

## Results and Discussion

### Characterization of PBMLR NPs

**Figure 1:**
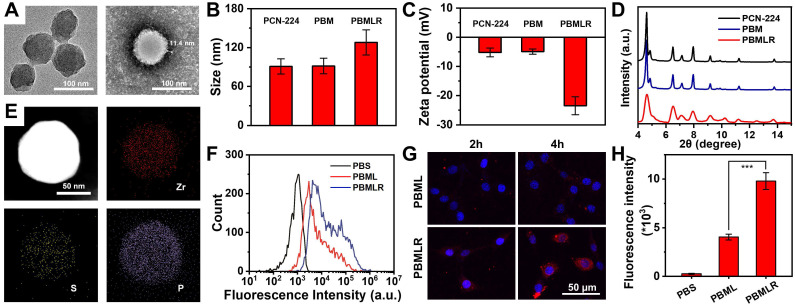
Schematic and characterization of PBMLR NPs. (A) TEM images of PCN-224 (left) and PBMLR NPs (right). (B) Hydrodynamic diameter and (C) Zeta potential of PCN-224, PBM, and PBMLR NPs. (D) PXRD of PCN-224, PBM, and PBMLR NPs. (E) Energy-dispersive X-ray spectroscopy mapping of the PBMLR NPs. The presence of Zr indicates the PCN-224 nanostructure, while S indicates BSO and P indicates the lipid bilayer. (F) Flow cytometry analysis for the binding or internalization of the different nanoparticle formulations at the concentration of 1 mg mL^-1^ after 4 h incubation with 4T1 cells. (G) Confocal microscopy images of 4T1 cells after 2 h and 4 h incubation with PBML and PBMLR NPs at the concentration of 1 mg mL^-1^. NPs were stained with DiD (red) and the nucleus was stained with DAPI (blue). (H) The average cellular fluorescence signal of each group in (B). (n = 5). *** *P* < 0.001.

Zirconium-based porphyrinic framework nanoparticles PCN-224, as excellent photosensitizers, which can load inhibitor molecules with high loading efficiency 21,22, were synthesized as precursors through a one-step hydrothermal method 20. The photosensitizer content of H2TCPP in PCN-224 was 54.4% (w/w) as determined via UV-Vis spectrophotometry, which guarantees the potential for efficient photodynamic reaction (Figure S1). To ensure a better EPR effect 23, the prepared PCN-224 nanoparticles were spherical with a controllable average diameter of approximately 90 nm, as shown in the TEM image (Figure 1A). To achieve MPI, two hydrophilic drugs, Met and BSO, as inhibitors of the critical pathway, were loaded into the PCN-224 nanoparticles. The co-loading efficiencies were measured as 29.1% and 24.7%, respectively, using inductively coupled plasma mass spectrometry (ICP-MS). Spherically shaped and negatively charged PBMLR NPs with a diameter of 120 nm were synthesized by coating a cRGD peptide-grafted lipid bilayer onto the PCN-224 cores (Figure 1A-C) to guarantee a long-circulation time and tumor-targeting abilities via intravenous administration 18,24. The elemental distribution within the PBMLR NPs, according to energy-dispersive X-ray spectroscopy (EDS), revealed a heavier deposition of phosphorus (P) in the peripheral layer compared to the deeper core. At the same time, zirconium (Zr) and sulfur (S) were uniformly distributed within the core region, implying the formation of a lipid bilayer bound to BSO in the PCN-224 cores (Figure 1E). Furthermore, no significant crystalline phases or photosensitivity changes in the PCN-224 and PBMLR nanoparticles were measured via X-ray diffraction (XRD) and fluorescence of singlet oxygen sensor green (SOSG) (Figure 1D and Figure S1), confirming that the lipid coating retains the performance of PCN-224 as both photosensitizer and drug vehicle. No significant aggregate or size changes of PBMLR NPs could be observed after incubation in PBS (pH = 7.4) for a week and culture medium for two days (Figure S2), demonstrating good stability beneficial for long blood circulation.

The release rate of BSO and Met reached 55.6% and 60.5% within 24 h under acidic conditions (pH = 5.5, simulating the pH in the lysosomes of tumor cells), and the release rate at pH = 7.4 was 9.1% and 9.9%, suggesting the negligible drug leakage during long-term circulation and less potential risks to normal tissues (Figure S3). No significant difference was observed in the release rate between Met and BSO, suggesting these two drugs may be simultaneously released from PBMLR in response to the acidic microenvironment of the lysosome 25.

To exclude the potential cytotoxicity of the PBMLR NPs themselves, no cytotoxic dose level was measured for cultured cells. The nontoxic concentrations of Met and BSO (survival rate > 95%) were calculated to be 2.3 × 10^-4^ M and 1.1 × 10^-4^ M, respectively, and there was no obvious combined toxic effect below the indicated doses (Figure S4). In addition, the cytotoxicity of PCN-224 or PBMLR was negligible at concentrations below 100 mg L^-1^ (Figure S5). The PBMLR NPs used in the present study did not exceed these conditions.

As critical metabolic pathway inhibitors, the direct targets of Met and BSO were complex I in mitochondria and γ-GCS in the cytoplasm, respectively, suggesting that the cellular uptake of the nanoplatform is an essential prerequisite for MPI. Given the weak fluorescence signals of PBMLR NPs (Figure S1), the red fluorescent dye DID was used to label the lipid bilayer to characterize the cellular uptake and intracellular behavior of the PBMLR NPs. Using the confocal laser scanning microscopy (CLSM) and flow cytometry (FCM), a 2.4-fold increased uptake of PBMLR was observed 4 h after co-incubation with 4T1 cells (Figure 1F-H) compared to PBML NPs (with no cRGD peptide modification), demonstrating that surface modification with cRGD peptide could optimize endocytosis mediated by the integrin α_v_β_3_ 26.

### Long-term modulation of cellular metabolism by MPI

**Figure 2:**
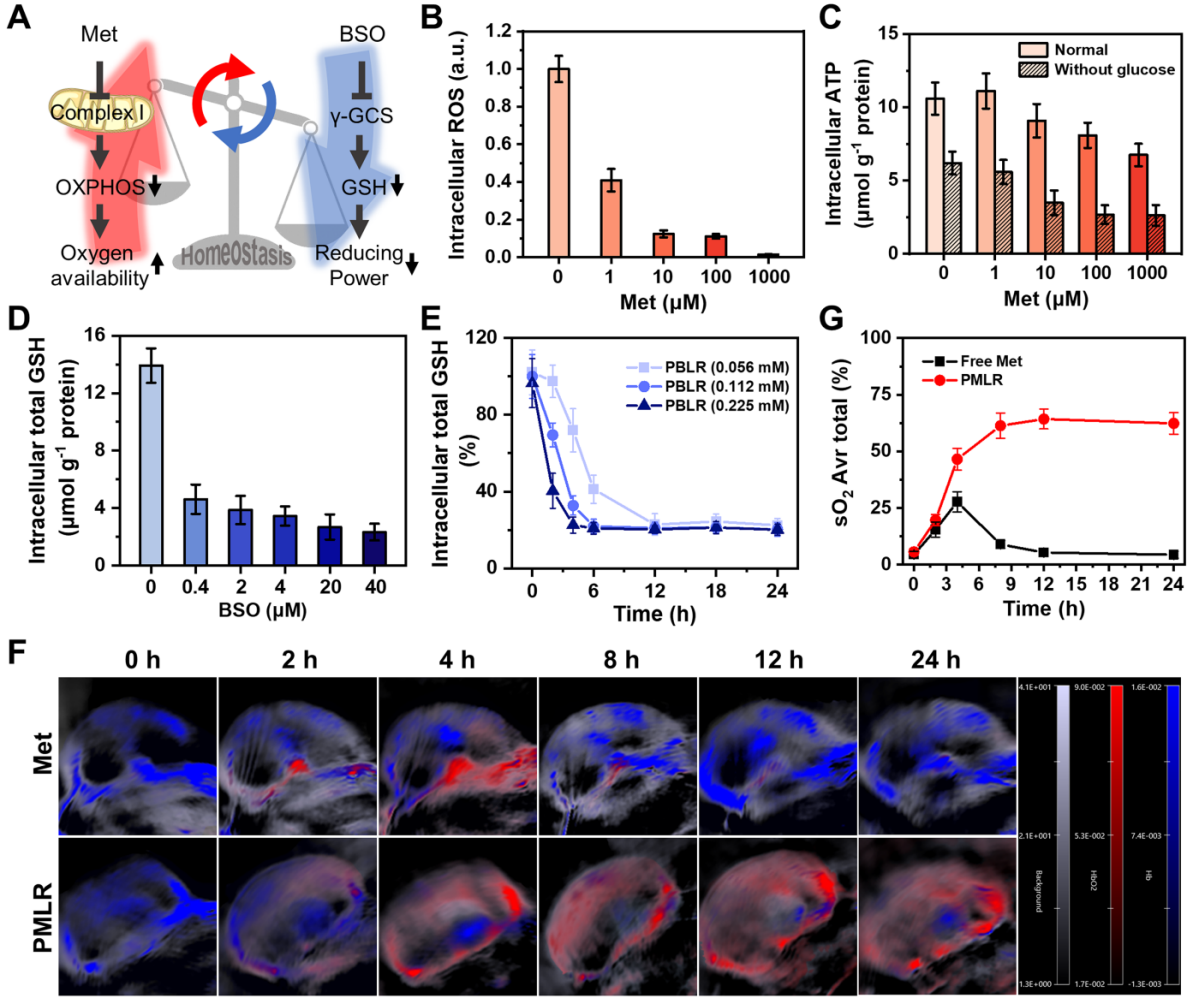
Long-term modulation of cellular metabolism by MPI. (A) Schematic illustrations of the effects of two inhibitors on cellular metabolism. (B) Intracellular ROS of 4T1 cells after incubation with free Met for 4 h under hypoxia without laser irradiation. (C) Intracellular ATP level in 4T1 cells by 4 h after incubation with free Met, as stained using an ATP Assay Kit based on firefly luciferase. Intracellular total GSH level in 4T1 cells 4 h after incubation with (D) free BSO and (E) PBLR NPs (BSO concentrations: 0.056 mM, 0.112 mM, and 0.225 mM) as determined by using a Total GSH Assay Kit based on DTNB. (F) PA images of 4T1 solid tumors at various time points, measuring deoxygenated hemoglobin (λ = 750 nm) and oxygenated hemoglobin (λ = 850 nm) after injection with free Met or PMLR (100 µL, 1 mg mL^-1^ Met). The color scale represents the blood oxygen saturation, calculated from the PA signal ratios between oxygenated and deoxygenated hemoglobin. The grayscale images are superimposed B-scan ultrasound images. (G) Quantification of the oxyhemoglobin saturation over time (oxygenated hemoglobin: total hemoglobin), across the total tumor area (sO_2_ Avr Total), using the PA imaging data shown in (F). The relative tumor oxygenation was determined from the PA signal ratios between oxygenated and total hemoglobin.

To verify the long-lasting effects of MPI, we first evaluated the effects of the two inhibitors on cellular metabolism *in vitro* (Figure 2A). When simulating the hypoxic tumor environment in deeper regions (1.5% oxygen) by a live-cell workstation, at least an 87.8% decrease in intracellular ROS was observed (Figure 2B and Figure S6) when the concentration of Met was above 10 μM. This confirms that Met efficiently inhibited mitochondrial complex I in electron transport 25, which is the major source of mitochondrial ROS 27. Besides, since glutamine-driven TCA cycle flux compensatory upregulation, oxidative phosphorylation (OXPHOS) remains the largest quantitative contributor to ATP production during hypoxia and glucose starvation 28. Cells showed decreased ATP levels after Met treatment (Figure 2C), confirming that Met induced the downregulation of OXPHOS and reduced metabolic oxygen consumption. As for the cells treated with BSO, there was a 5.0-fold decrease in total intracellular GSH, with a dose-dependent effect (Figure 2D), indicating the weakened redox systems based on GSH/GSSG due to the marked inhibition of biosynthesis. No obvious difference in the change of GSH was observed between cells in hypoxia or normoxia (Figure S7), showing that oxygen concentration did not affect BSO regulation.

However, metabolic interventions are unlikely to be sustained in the long term because of regulators' elimination 29. To investigate whether PBMLR could have a long-term effect on metabolism, the regulation of GSH biosynthesis was first measured after treatment with PBLR (BSO-loaded) *in vitro*. We found that an increased BSO dose led to more rapid GSH downregulation, while an extremely small BSO dose could downregulate GSH levels by more than 80% within 2 h and maintain it for up to 24 h (Figure 2E). In contrast, the regulation of aerobic cell metabolism by Met cannot be sustained because of its reversible non-competitive inhibition mechanism *in situ* in the respiratory chain to avoid the side effects on normal cells, causing the risk of lactic acidosis. Fortunately, a sustained release owing to encapsulation by PCN-224 NPs has a great potential in prolonging the effect of Met, which was verified in a mouse model of subcutaneous tumors. Mice bearing 4T1 tumors with a volume of approximately 100 mm^3^ were intravenously injected with either free Met (100 µL, 1 mg mL^-1^) or PMLR (PBMLR without BSO; 100 μL, equal to 1 mg mL^-1^ Met), and oxyhemoglobin saturation was measured using a Vevo LAZR Imaging System (FujiFilm VisualSonics Inc.) (Figure 2F). Tumor oxygenation increased 4.6-fold and 8.4-fold in 4 h post-injection with free Met and PMLR, respectively. However, intratumoral oxyhemoglobin saturation in mice injected with free Met decreased over time, most likely because of the rapid metabolism and elimination. Conversely, mice injected with PMLR NPs showed sustainedly increased tumor oxygenation, from 5.6% to 64.3%, which indicated the long-lasting effect of Met intervention. This is mainly due to the enhanced tumor uptake and continuous Met release of PMLR NPs (Figure 2G).

Such low-dose, long-lasting modulation is interesting because it helps create a larger treatment window for subsequent therapies. Thus, the co-delivery of Met and BSO through PBMLR was considered to be a viable approach to achieving efficient long-term metabolic modulation. We expect that this metabolic pre-intervention (MPI) strategy could play a synergistic effect in ROS-mediated dynamic therapy.

### Efficient and synergistic modulation of cellular metabolism by MPI

Furthermore, we studied how MPI efficiently affects the sensitivity of cells to photo-induced ROS, which is reflected in the intracellular accumulation of ROS triggered by low-dose PDT. The light-condition screening experiment was conducted by evaluating the cytotoxicity under different light doses and at different drug doses (Figure S8). Finally, we determined that the PBMLR NPs at a concentration of 50 mg L^-1^ under a light dose of 20 mW cm^-2^ for 15 min did not exhibit phototoxicity under normoxic conditions.

**Figure 3:**
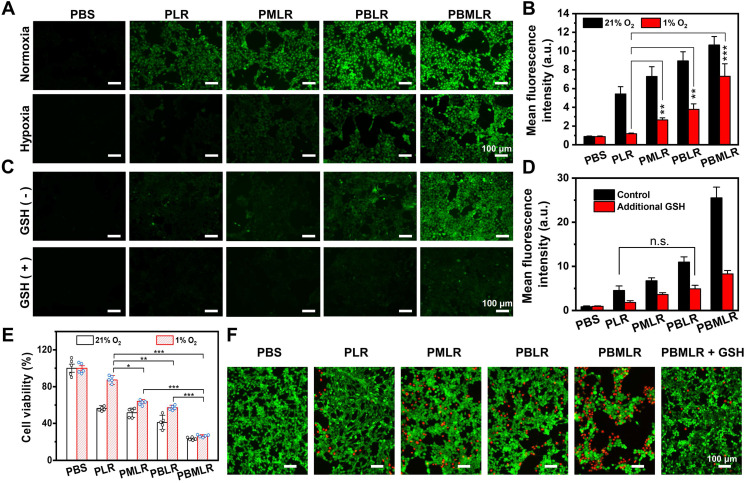
Efficient and synergistic modulation of cellular metabolism by MPI for PDT enhancement. (A) 4T1 cells were cultured in a hypoxic environment to simulate the microenvironment in solid tumors. Intracellular ROS accumulation (green fluorescence) was determined by using an inverted fluorescence microscope. 4h after incubation under normoxic or hypoxic conditions, the cells were stained with DCFH-DA and then irradiated. Scale bar: 100 µm. (B) Quantitative analysis of fluorescence intensity in (A) using the analysis software ImageJ. (C) Intracellular ROS accumulation (green fluorescence) was determined by using an inverted fluorescence microscope. Four hours after incubation under hypoxic conditions or the addition of GSH, the cells were stained with DCFH-DA and then irradiated. Scale bar: 100 µm. (D) Quantitative analysis of fluorescence intensity in (C) using the analysis software ImageJ. (E) The viability of 4T1 cells was measured using the MTT assay. The cells were incubated with low concentrations of PLR, PMLR, PBLR, and PBMLR NPs and irradiated under the conditions described above. (F) Live/dead cell staining 4 h after treatment and laser irradiation. Scale bar: 100 µm. The results are expressed as the mean ± SD (n = 5). * *P* < 0.05, ** *P* < 0.01, and *** *P* < 0.001 denote statistical significance assessed by using one-way ANOVA. n.s. denotes no significant difference.

The intracellular ROS-generating capability under laser irradiation was identified based on the fluorescence intensity of the ROS-sensitive probe 2′,7′-dichlorofluorescein diacetate (DCFH-DA) under both normoxic and hypoxic conditions. Under normoxic conditions, the fluorescence intensity of the PMLR NPs was similar to that of the PLR NPs without Met loading (Figure 3A), which could be explained by the ample oxygen availability in the cells during treatment. Meanwhile, the amount of ROS generated by the PLR and PMLR NPs was slightly lower than that generated by the PBMLR and PBLR NPs, suggesting a positive impact of BSO intervention on ROS accumulation. The normalized signal intensities from the fluorescence of DCFH-DA analyzed using flow cytometry were consistent with the images (Figure 3B). Encouragingly, the PMLR NPs exhibited markedly increased DCFH-DA fluorescence intensity (2.6-fold) compared to the control group under hypoxic conditions, demonstrating that Met can alleviate hypoxia and increase ROS generation within PSs upon laser irradiation. Besides, we observed that oxygen availability provided by PMLR NPs (only Met) could only affect a ROS generation of 42.9%, while GSH downregulation by PBLR NPs (only BSO) compensated for 67.9%. This means that neither PMLR nor PBLR NPs could compensate for the shortcomings of relatively low oxygen content under hypoxic conditions. Intriguingly, the PBMLR NPs exhibited 130.9% ROS accumulation, exceeding the normoxic control group, suggesting that Met and BSO may work synergistically to overcome the adverse intracellular environment.

To further prove whether PBMLR can overcome the ROS scavenging caused by redox systems, additional GSH of physiological concentration was added to emulate the enhanced compensatory pathway as a control to shield the effect of BSO. The addition of exogenous GSH markedly decreased DCFH-DA fluorescence intensity under hypoxic conditions (Figure 3C), suggesting significantly lower levels of ROS accumulation. However, GSH did not affect the trend of differences in ROS accumulation between different groups, which was consistent with the quantitative results obtained via flow cytometry (Figure 3D). However, neither PBLR intervention nor Met/BSO co-intervention with PBMLR cannot break through this shielding effect, proving that the BSO intervention strategy downregulates GSH through cellular metabolic consumption with suppressed biosynthesis rather than directly consuming GSH. From another viewpoint, PBMLR intervention combined with Met effectively alleviated the consumption of redox buffer systems by ROS. This also reflects a potential mechanism for the efficient and long-lasting modulation of metabolic homeostasis, owing to the synergistic effect of critical pathway inhibitors.

### PDT enhancement *in vitro* by MPI

To further prove that MPI by PBMLR NPs can improve PDT, photocytotoxicity was measured using the MTT assay (Figure 3E and Figure S9). Cells treated with PBMLR NPs under a light dose of 50 mW cm^-2^ for 15 min exhibited half-lethal phototoxicity. The PBMLR NPs had 4.2-fold stronger phototoxicity than the PLR NPs based on cell viability. Moreover, the PMLR and PBLR NPs showed 2-fold and 1.5-fold stronger killing capacity, respectively, than PLR NPs. MPI by PBMLR NPs showed a statistically synergistic effect on cell killing, owing to a similar effect on ROS accumulation. Compared with normoxic conditions, PLR NPs exhibited only one-fifth of their lethality under hypoxic conditions, which coincided with hypoxia tolerance in clinical practice. To verify whether the loading ratio of Met and BSO has an impact on the therapeutic outcome, different loading ratios of 1:1, 1:2, 1:4, and 1:9 were measured under conditions of hypoxia. As shown in Figure S10, significantly enhanced photocytotoxicity could be observed when the loading ratio of Met and BSO is close to 1:1. This optimal content ratio of Met and BSO was chosen for the further therapeutic experiment.

The results of live/dead cell staining using Calcein-AM/PI Staining Kit were similar to those described above to evaluate the PDT under hypoxia with a non-lethal light dose of 20 mW cm^-2^ for 15 min (Figure 3F). Cells incubated with PBS and PLR NPs under laser irradiation exhibited only green fluorescence, indicating poor phototoxicity of these treatments. Cells incubated with the PMLR, PBLR, and PBMLR NPs exhibited mostly green fluorescence and slight red fluorescence, indicating enhanced toxicity under hypoxic conditions after laser irradiation. Cells incubated with the PBMLR NPs and additional GSH exhibited less red fluorescence than those incubated with PBMLR NPs, indicating that the PBMLR NPs exhibited high lethality due to the downregulation of redox buffer systems by MPI. Strikingly, PBMLR NPs exhibited the same lethality under both hypoxic and normoxic conditions, indicating that MPI may theoretically overcome the resistance of the intracellular environment to cytotoxic ROS mediated by PDT.

### Anticancer effect of enhanced PDT after MPI *in vivo*

**Figure 4:**
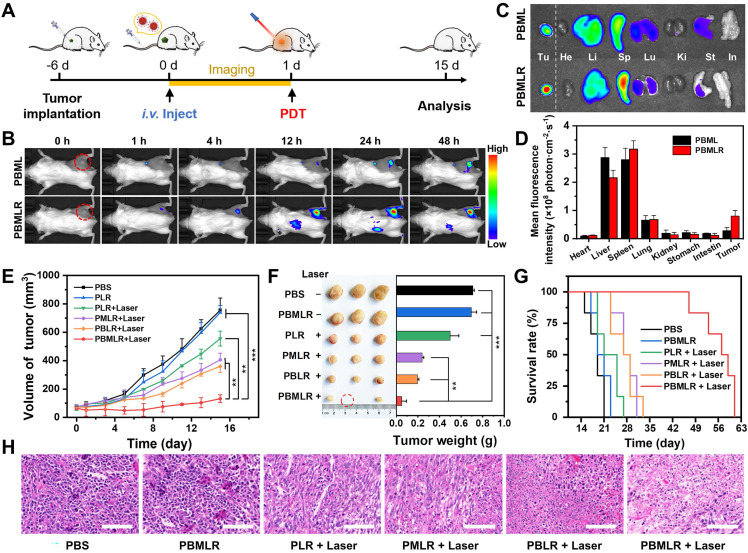
*In vivo* antitumor effect of MPI based on PBMLR. (A) Imaging and therapeutic schedule for PBMLR NPs. Tumor-bearing mice were intravenously injected with equal concentrations of MOF nanoparticles (100 µL, 2 mg mL^-1^) and subjected to 15 min of laser irradiation 24 h post-injection (50 mW cm^-2^). (B) *In vivo* fluorescence images of 4T1 tumor-bearing mice taken at different time points post-injection with PBML and PBMLR. Tumor sites are circled in red. (C) *Ex vivo* fluorescence images of the major organs (heart, liver, spleen, lungs, and kidneys) and tumors at 24 h post-injection. (D) Quantitative analysis of the PBMLR fluorescence intensity of the organs and tumors *ex vivo* using the analysis software ImageJ. (E) Tumor growth curves following different treatments. Shown are data from the PBS and PBMLR NPs-treated mice, and mice that received PDT 24 h after injection with PLR, PBLR, PMLR, and PBMLR. (F) Tumor weights and representative images of the excised tumors after the different treatments. (G) The 9-week survival curve after the different treatments. (H) H&E staining of tumor tissues after the different treatments. Scale bar: 100 µm. The significance between each pair of groups was calculated using Student's t-test. * *P* < 0.05, ** *P* < 0.01, *** *P* < 0.001. The values are presented as the mean ± SD, n = 6.

The *in vivo* behavior of the PBMLR NPs was then investigated by labeling the lipid bilayer with the red fluorescent dye DIR. The biodistribution and tumor imaging of PBMLR were determined at 1, 4, 12, 24, and 48 h post-injection (Figure 4A). Fluorescent signals were distinctly observed in tumor areas 1 h after an intravenous injection of the PBMLR (200 µL, 1 mg mL^-1^) using an FX Pro *in vivo* fluorescence imaging system (Figure 4B). In addition, PBML NPs (without cRGD modification) were observed in tumors later than PBMLR, at 24 h post-injection. To accurately determine the biodistribution, we further performed imaging of the main organs at the time points mentioned above. Despite being trapped in the mononuclear phagocyte system with a similar organ distribution (Figure 4C), the high fluorescence intensity in tumors was analyzed. It showed a 12% total enrichment of the PBMLR (Figure 4D) and a 3-fold increase over the PBML treatment in either speed or accumulation (Figure S11), benefitting from the tumor neovascularization targeting of the cRGD peptide, indicating that PBMLR could potentially be used for tumor-targeted imaging and therapy.

Confirming the accumulation of PBMLR NPs in tumors, the anticancer effect of MPI was explored in subcutaneous tumor mouse models (Figure 4A). Mice bearing 70 mm^3^ 4T1-tumors were divided into six groups: (1) control (PBS), (2) PBMLR, (3) PLR plus laser irradiation (50 mW cm^-2^, 15 min) (4) PMLR plus laser irradiation, (5) PBLR plus laser irradiation, and (6) PBMLR plus laser irradiation, each of which was intravenously injected with the MPI modulator (100 μL, equal to 2 mg mL^-1^ PCN-224, 1.26 mg mL^-1^ Met, and 1.07 mg mL^-1^ BSO). After 24 h, the tumor areas were irradiated at a light dose of 50 mW cm^-2^ for 15 min, and the mice were observed for another 14 days.

Compared to the PBS group, the PBMLR group exhibited only a slight tumor inhibition effect under laser irradiation, whereas the group of only PBMLR had no effect (Figure 4E). In sharp contrast, significant anti-tumor effects were observed in the PBLR, PMLR, and PBMLR groups upon laser irradiation, among which the PBMLR plus laser irradiation group exhibited maximum tumor growth inhibition. Notably, the PBMLR plus laser irradiation group was still able to show signs of long-lasting tumor growth control compared to the PBLR and PMLR groups at nine days post-PDT (Figure 4E), suggesting the synergistic antitumor effect of the MPI strategy. The significantly extended survival in the PBMLR plus laser irradiation group supports these results (Figure 4F). Representative images of hematoxylin and eosin (H&E) staining showed more necrotic regions in the PBMLR plus laser irradiation group, further confirming the therapeutic efficacy of the NPs (Figure 4G). In addition, both negligible changes in body weight and non-obvious lesions in the major organs implied the good biocompatibility of MPI based on PBMLR NPs (Figures S12 and S13).

## Discussion

An abnormal intracellular environment is a metabolic adaptation of tumor cells in response to metabolic stress during tumor progression 25. Herein, we proposed an MPI strategy based on the long-lasting inhibition of critical metabolic pathways. A co-delivery system was established using a porphin-MOF nanoplatform loaded with the metabolic regulators Met and BSO for enhanced anti-tumor PDT. The obtained biocompatible PBMLR NPs accumulated in the tumor tissue after intravenous injection due to the accelerated uptake and targeting ability of the cRGD peptide. Met, which was steadily released in the acidic lysosome environment and acted on the mitochondria, hindering mitochondrial respiration and guaranteeing oxygen availability. At the same time, BSO targeted γ-GCS and inhibited GSH biosynthesis, weakening the detoxification pathway for eliminating ROS. Met and BSO synergistically rebuilt a new metabolic adaptation system, thus contributing to the efficient and long-lasting modulation of the cellular metabolic environment.

This substrate-independent regulation could effectively circumvent the compensatory accessory pathway that the cell self-adaptively initiates in response to internal and external metabolic stress 30,31, ensuring the sustainability of metabolic regulation in the temporal dimension. This strategy highlights the significance of reasonable inhibitor screening, which involves many aspects of a drug's metabolic pathway, pharmacodynamics, and physiological toxicity. Ordinarily, it is difficult for a single inhibitor to cover such conditions comprehensively. For instance, Met has low cytotoxicity but is coupled with a risk of lactic acidosis. However, it has milder but more controllable effects than other common respiratory chain inhibitors like phenformin and rotenone 32,33. In addition, using inappropriate inhibitors may force cells to establish new adaptations against the artificial metabolic regulation we imposed. This may lead to low metabolic efficiency, such as the compensation of OXPHOS flux by the classic glutamine-dependent acetyl-CoA formation pathway 34.

## Conclusions

In summary, we developed a simple, safe, and effective photodynamic nano-agent, PBMLR, for enhanced anti-tumor PDT using a metabolic pre-intervention strategy. The porphyrinic MOF nanostructure of PBMLR was conducive to its tumor-targeting capability and was optimized *in vivo* for use simultaneously as a PDT agent and drug delivery vehicle. The small molecule inhibitors Met and BSO were used to modulate the unfavorable tumor metabolic environment by pre-intervening in critical cellular metabolic pathways, and it should be a much simpler alternative to existing methods such as the in-situ generation of oxygen or the elimination of GSH by delivering exogenous oxidants. Along with this aspect, the synergistic effect of metabolic modulation resulted in long-lasting intracellular environment remodeling, providing a wide time window for PDT and producing powerful anticancer PDT effects. Aside from long-lasting interventions on the critical metabolic pathway to bypass the “anti-ROS” compensatory pathway, we believe this nanosystem may also be combined with other nanotechnology-based approaches to optimize anticancer therapies that utilize ROS in the cell-killing process, such as radiodynamic therapy and sonodynamic therapy. Furthermore, the MPI strategy we presented here can be a potential strategy for diagnosing and treating other diseases in which metabolic adaptation exists.

## Supplementary Material

Supplementary figures.
