# Supplementary material for: Redox dyshomeostasis modulation of the tumor intracellular environment through a metabolic intervention strategy for enhanced photodynamic therapy
Source: Theranostics. 2022 Aug 15;12(14):6143–54. doi: 10.7150/thno.75837 (PMC9475470; doi:10.7150/thno.75837)
Supplement: Supplementary file 1 — Supplementary figures. [file thnov12p6143s1.pdf]

Supporting Information

Redox dyshomeostasis modulation of the tumor intracellular environment through a metabolic intervention strategy for enhanced photodynamic therapy

Zuo Yang, Chaoqiang Qiao, Qian Jia, Zhuang Chen, Xiaofei Wang, Xuelan Liu, Ruili Zhang*, Kanyi Pu*, Zhongliang Wang*

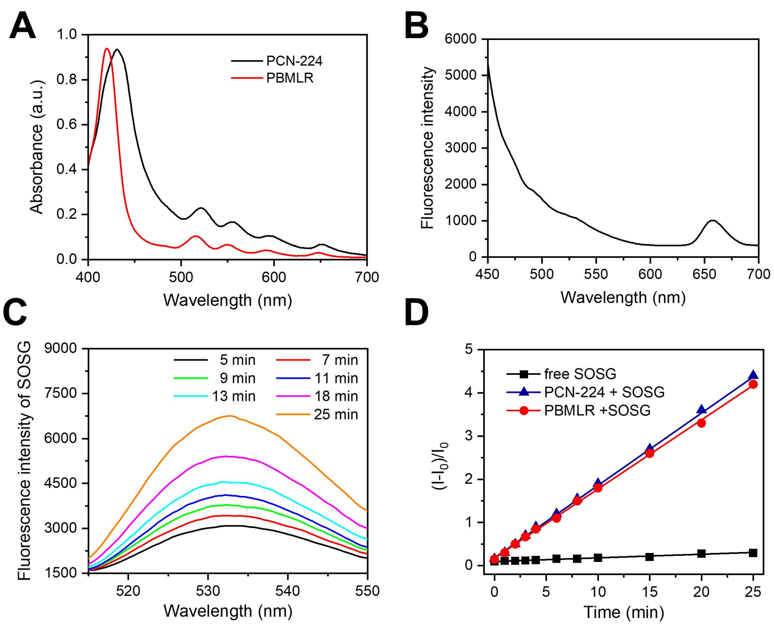

Figure S1. UV-Vis absorption, fluorescence spectra, and photosensitivity characterization: (A) UV-Vis absorption of PCN-224 and PBMLR NPs. (B) Fluorescence spectra of PBMLR NPs. (C) Fluorescence spectra of SOSG incubated with PBMLR NPs. (D) Comparison of fluorescence intensity changes of SOSG incubated with PBS, PCN-224, and PBMLR.

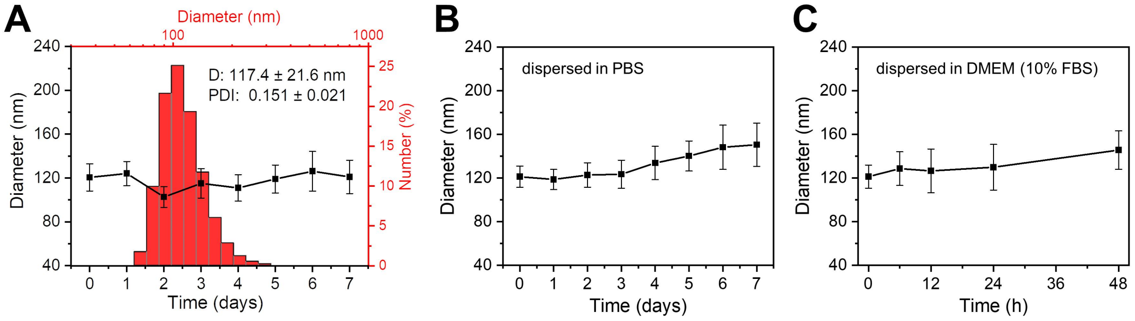

Figure S2. Stability of PBMLR NPs: (A) Size changes and the average diameter of PBMLR NPs in PBS (pH 7.4). (B) Size changes of PBMLR NPs in PBS. (C) Size changes of PBMLR NPs in culture medium with 10% FBS. The values are presented as the mean ± SD, n = 5.

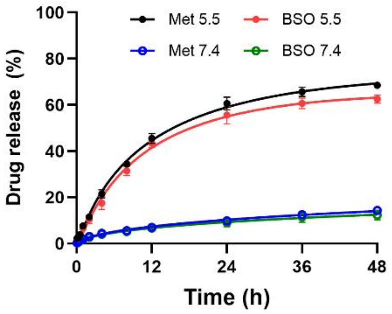

Figure S3. Relative Met and BSO release from PBMLR examined in PBS solution with different pH at 37 °C.

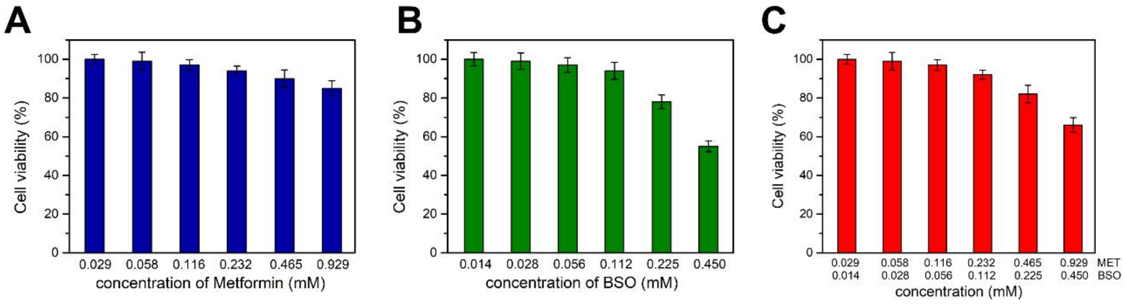

Figure S4. Cell viability of 4T1 cells 24 h after treatment with (A) Met, (B) BSO, and (C) both Met and BSO at different concentrations. The values are presented as the mean ± SD, n = 5.

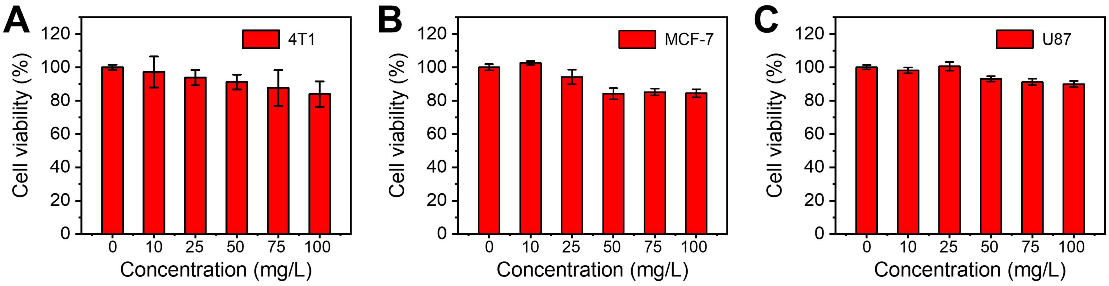

Figure S5. No observed adverse effect of PBMLR on different tumor cell lines: Cell viability of (A) 4T1 cells, (B) MCF-7 cells, and (C) U87 cells 24 h after treatment with PBMLR NPs without irradiation. The values are presented as the mean ± SD, n = 5.

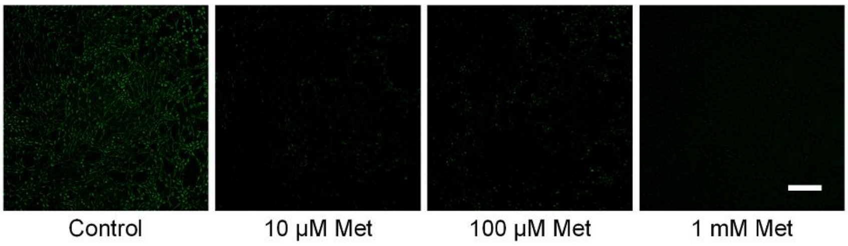

Figure S6. Imaging intracellular oxidative level in 4T1 cells treated with different concentrations of Met. Under hypoxic conditions, cells were incubated with DCFH-DA and intracellular ROS (green fluorescence) was immediately determined using an inverted fluorescence microscope. Scale bar: 100 μm.

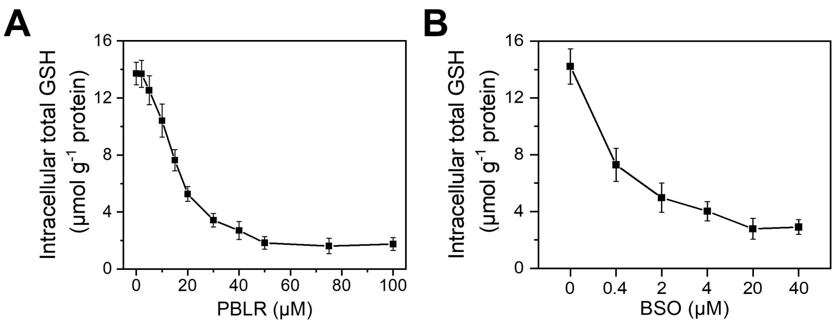

Figure S7. Intracellular total GSH levels in 4T1 cells by 4 h after incubation with (A) PBLR (equal concentration of BSO) under normoxic conditions and (B) BSO under hypoxic conditions. The values are presented as the mean ± SD, n = 5.

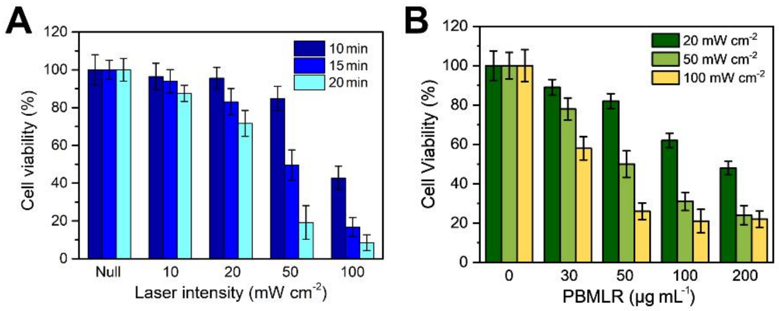

Figure S8. The light condition screening experiment: (A) Viability of 4T1 cells 12 h after treatment with PBMLR NPs at the concentration of 50 μg mL^{-1} under different laser irradiation. (B) Viability of 4T1 cells 12 h after treatment with PBMLR NPs at different concentrations under 15 min laser irradiation. The values are presented as the mean ± SD, n = 5.

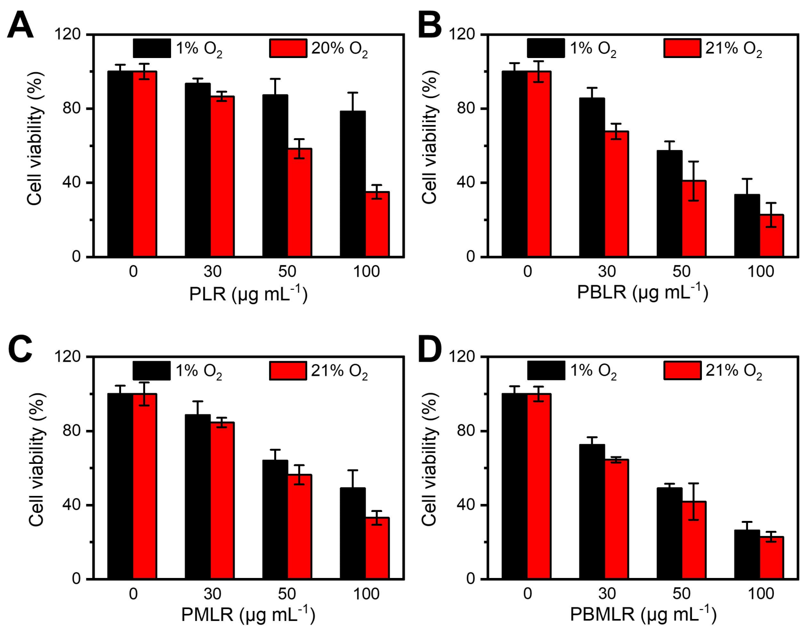

Figure S9. The viability of 4T1 cells was measured using the MTT assay. 4T1 cells were cultured with (A) PLR NPs, (B) PBLR NPs, (C) PMLR, and (D) PBMLR under normoxic or hypoxic conditions for 12 h, and then were irradiated under a light dose of 50 mW cm^{-2} for 15 min, and further cultured for 12 h. The values are presented as the mean ± SD, n = 5.

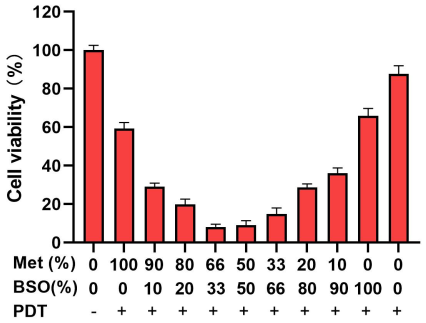

Figure S10. Viability of 4T1 cells 12 h after PDT treatment with 50 μg mL^{-1} PBMLR NPs with different loading content ratios of Met and BSO under hypoxia conditions. The values are presented as the mean ± SD, n = 5.

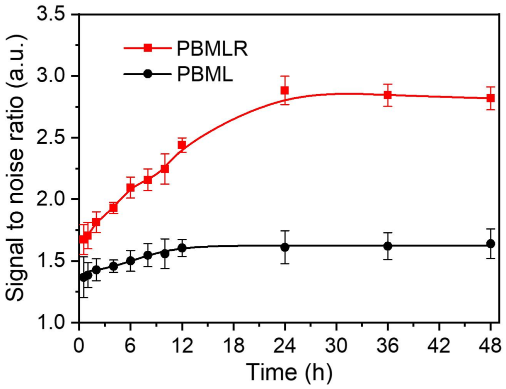

Figure S11. Signal to noise ratio based on fluorescence intensity of PBML or PBMLR nanoparticles in the tumor region. Five-point sample method was used for semi-quantitative analysis with the software ImageJ according to fluorescence intensity results from IVIS. The values are presented as the mean ± SD, n = 5.

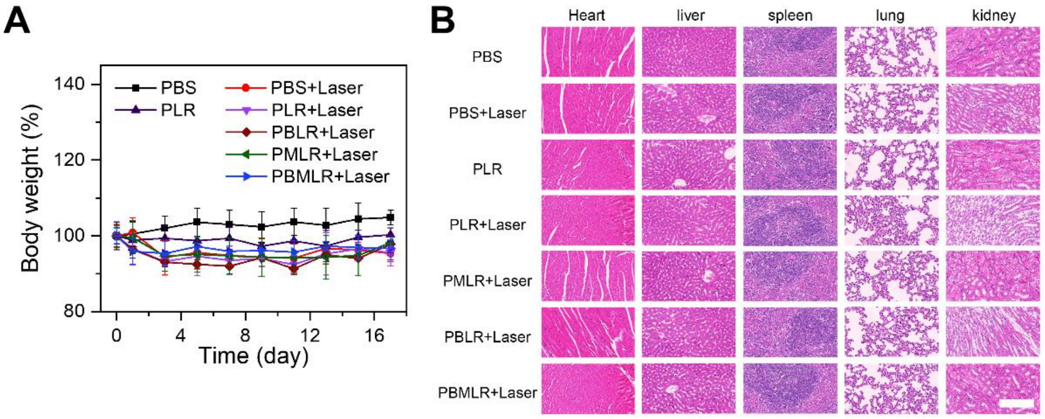

Figure S12. Biocompatibility evaluation: (A) Body weights of mice after different treatments. (B) H&E staining of heart, liver, spleen, lung, and kidney in the mice from various groups. Scale bar: 100 μm.

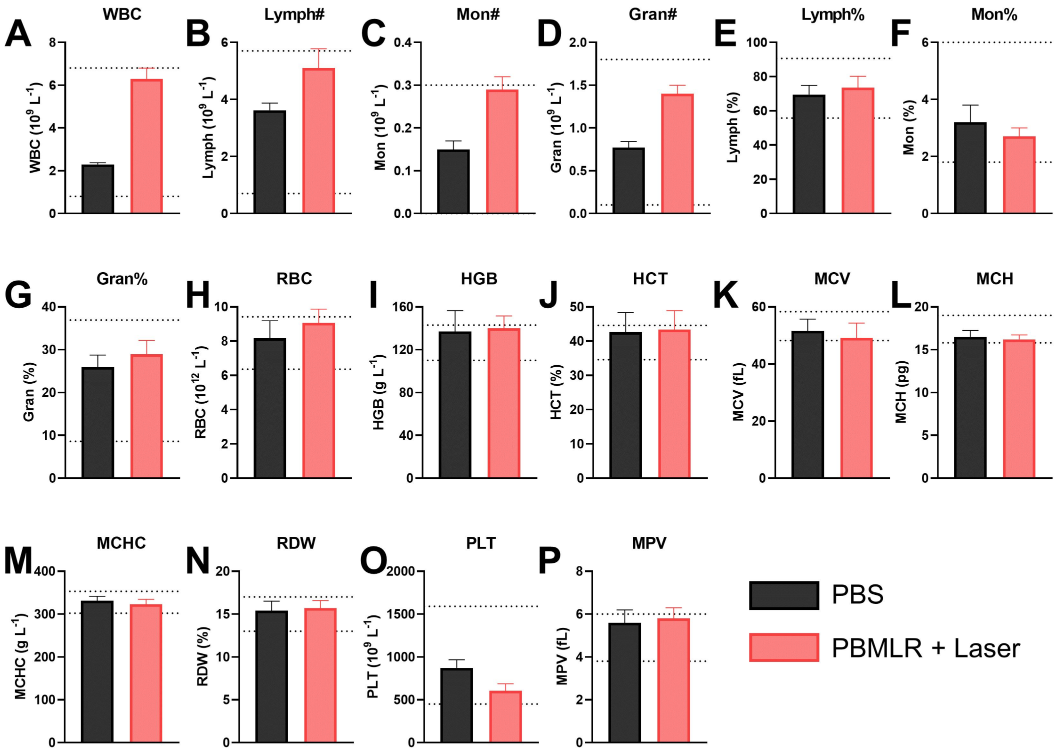

Figure S13. Blood biochemical tests of BALB/c mice treated with PBMLR-based PDT. The data were collected 24 h after PDT: (A) White blood cells (WBC). (B) lymphocyte (Lymph#). (C) monocytes (Mon#). (D) neutrophils (Gran#). (E) percentage of lymphocytes (Lymph%). (F) percentage of monocytes (Mon%). (G) percentage of neutrophils (Gran%). (H) red blood cells (RBC). (I) hemoglobin (HGB). (J) hematocrit (HCT). (K) mean corpuscular volume (MCV). (L) mean corpuscular hemoglobin (MCH). (M) mean corpuscular hemoglobin concentration (MCHC). (N) red blood cell distribution width (RDW). (O) platelets (PLT). (P) mean platelet volume (MPV). All of these parameters were within the normal range (black dotted line). The values are presented as the mean ± SD, n = 3.
